# Cost-effectiveness of chronic fatigue self-management versus usual care: a pilot randomized controlled trial

**DOI:** 10.1186/s12875-014-0184-7

**Published:** 2014-11-25

**Authors:** Hongdao Meng, Fred Friedberg, Melissa Castora-Binkley

**Affiliations:** School of Aging Studies, University of South Florida, 13301 Bruce B Downs Blvd., MHC 1300, Tampa, FL 33620 USA; Department of Psychiatry, Stony Brook University Medical Center, Department of Psychiatry and Behavioral Science, Putnam Hall/South Campus, Stony Brook, NY 11794 USA

**Keywords:** Cognitive behavioral therapy, Cost-effectiveness, Fatigue

## Abstract

**Background:**

Fatigue is a common yet difficult to treat condition in primary care. The objective of this study is to evaluate the cost-effectiveness of a brief cognitive behavioral therapy (CBT) based fatigue self-management (FSM) intervention as compared to usual care among patients with chronic fatigue in primary care.

**Methods:**

An economic evaluation alongside of a parallel randomized controlled study design was used. Computer-generated variable-sized block randomization plan was used to assign patients into treatment groups and data collection staff were blinded to group assignments. Patients aged between 18 and 65 years with at least six months of persistent fatigue and no medical or psychiatric exclusions were enrolled from a large primary care practice in Stony Brook, New York. The FSM group (n = 37) received two sessions of a nurse-delivered, fatigue self-management protocol and a self-help book and the usual care group (n = 36) received regular medical care. The effectiveness measure was the Fatigue Severity Scale and the cost measure was total health care expenditures derived from monthly health services use diaries during follow-up. A societal perspective was adopted and bootstrapped incremental cost-effectiveness ratios (ICERs) and net monetary benefit (NMB) were calculated as measures of cost-effectiveness.

**Results:**

The ICER for FSM was -$$2358, indicating that FSM dominates UC and it may generate societal cost savings as compared to usual care. Complete case analysis yielded smaller ICER (−$1199) with greater uncertainties. Net monetary benefit analysis showed that FSM has a probability of 0.833 (95% CI: 0.819, 0.847) to achieve positive NMB and the favorable results were not sensitive to assumptions about informal care or treatment costs.

**Conclusion:**

This economic evaluation found initial evidence that a two-session brief CBT-based FSM may be cost-effective as compared to usual care over 12 months. The FSM intervention is potentially a promising intervention for chronic fatigue patients in primary care. Additional research is needed to examine the reproducibility and generalizability of these findings.

**Trial registration:**

ClinicalTrials.gov (NCT00997451, March 28, 2009).

## Background

Chronic fatigue is characterized by persistent and recurring fatigue that cannot be alleviated by rest [1,2]. It has been associated with lower quality of life [3-5] and higher health care utilization [6-10]. The cost of lost productivity associated with chronic fatigue has been estimated to be between £75-£129 million annually in the U.K. and the burden of disease was especially high among those who access specialist services [11].

Cognitive behavioral therapy (CBT) has been shown to be effective in reducing fatigue symptoms as compared to adaptive pacing therapy or usual care [12], while graded exercise therapy (GET) has seen mixed results [13-16]. CBT combines elements of both behavioral therapy and cognitive therapy to facilitate the identification and reduction of negative thoughts and to build activity tolerance and positive coping skills among chronic fatigue patients. However, economic evaluations for the treatment of chronic fatigue in primary care have been limited and the findings are generally inconclusive [17-20]. In addition, no economic evaluation study has been reported in the US. The assessment of the relative value of CBT will be important for programmatic and policy decisions that must balance costs and outcomes of care [21,22].

The purpose of the present study is to compare the cost-effectiveness of a brief CBT-based Fatigue Self-Management (FSM) intervention and usual care (UC) conditions in a sample of primary care patients with chronic fatigue.

## Methods

### Chronic fatigue self-management study

The Chronic Fatigue Self-Management Study is a randomized controlled trial involving 111 primary care patients with chronic fatigue in New York between 2009 and 2011. Details of the study and results of the primary end point were reported elsewhere [23]. While the study was powered for the primary outcome of fatigue impact on functioning, the economic evaluation was designed as a pilot and feasibility study. All patients were recruited from a family medicine/primary care practice with 14 attending physicians and 21 family practice residents. The inclusion criteria for participants were (a) between 18 and 65 years of age; (b) at least six months of persistent fatigue with no medical or psychiatric exclusions (as determined by primary care physicians and a psychiatric nurse). Exclusion criteria were: (a) Medical: fatigue due to identifiable medical conditions (such as autoimmune diseases) or to medications (such as beta blockers); (b) Psychiatric: psychosis or dementia, alcohol or substance abuse, depression with melancholic or psychotic features, and anorexia nervosa or bulimia nervosa. These Axis I psychiatric diagnoses were identified from a nurse-conducted Structured Clinical Interview for DSM-IV (SCID) [24]. The study protocol received ethical approval from the Stony Brook University Institutional Review Board (IRB) and the drafting of this manuscript adheres to the CONSORT statement [25].

After written informed consent forms and baseline assessments were obtained, patients were randomly assigned to one of three groups as follows: CBT-based FSM (n = 37), attention control (AC) (n = 38), and usual care (UC) (n = 36). A variable-sized block randomization procedure was used to minimize potential selection bias. The study statistician generated the random allocation sequence, the principal investigator conducted the initial telephone interview, and a graduate student assigned participants to interventions. Data collection staff were blinded to the group assignment and sample size was chosen to ensure adequate power to detect treatment effect on the primary outcome. Additional details of the study have been reported elsewhere [23]. The CBT-based FSM group received two individual face-to-face fatigue self-management training sessions with a nurse (for up to 60 minutes) and a 61-page self-management booklet containing material assigned and discussed in the two sessions. This protocol was adapted from an efficacious 12-session CBT program for Chronic Fatigue Syndrome (CFS) [26]. Patients in the AC group received two sessions with a nurse therapist regarding emotional support and home-based self-monitoring of symptoms, affect, and stress. The AC group was designed to control for therapist attention and homework assignments so that potential placebo effects can be isolated from the FSM treatment effect. The UC group received no treatment beyond usual medical care. All three groups were assessed at baseline and 12-month follow-up [23]. For the purpose of this study, patients in the AC group were excluded because the attention control would be dominated by the control group as it requires higher costs (due to therapists’ attention) with no commensurate benefit.

### Outcome measures

The primary outcome was the Fatigue Severity Scale (FSS). The FSS was designed to measure the effect of fatigue on functioning. It is comprised of nine items rated on a 7-point Likert scale, where one indicates no impairment and seven indicates severe impairment. A one-point decrease on the FSS is considered clinically significant improvement. It is a validated scale for use in CFS with high internal consistency [27] and has been shown to be sensitive to treatment change [28].

### Service use and costs

Health resource use and costs were identified and valued from the societal perspective for the Reference Case analysis following the “Panel Recommendations” [29]. Health care resource use was measured with a modified version of the Client Service Receipt Inventory (CSRI), a validated health care utilization diary [30], to record health service use as well as informal care for the 3 month period prior to baseline and on a monthly basis by trained staff via a telephone interview during the post-treatment follow-up period.

To evaluate the economic effects of the prescribed treatments, we identified relevant cost categories of resource use by measuring utilization in each resource category (direct and indirect) and identifying the unit costs (prices) of the corresponding category. As economic endpoints, direct health care costs, direct non-health care costs and indirect costs were included [31]. The direct study-based health care costs included costs of the behavioral interventions and the economic consequences of the programs in terms of health services utilization before and after the intervention (direct health care costs). Intervention costs include costs of personnel (clinical psychologist, nurse interventionists, and staff), training, material (self-help booklet), time spent by study personnel and patients (intervention sessions and travel), facility costs (space, maintenance, and utilities), and other costs (advertising and telephone services). Costs were allocated to individual patients based on the number of sessions they attended. Direct health care costs included the costs of hospitalizations and visits to health care providers (e.g. general practitioner, specialist, physical therapist, alternative medicine providers) and the use of prescription and over-the-counter medications. The direct non-health care costs include out-of-pocket expenses, costs of paid and unpaid help, and travel costs of attending medical appointments. As part of the modified CSRI, information on the frequency of paid help, travel time for medical appointments, and the number of illness-related absences from paid or unpaid work were collected. Indirect costs include the value of production lost to society due to illness-related absence from work (paid or unpaid).

For each category of health care resources, we used standard approaches to estimate costs [32,33]. Unit costs for major health care services (e.g. provider office visits) and prescription medications were based on national average of Medicare payment rates, estimated from the 2010 Medical Expenditure Panel Survey (MEPS). Medicare payment rates are widely used as approximate measures of the opportunity costs associated with health services use in economic evaluations. Unit costs of various diagnostic tests were based on 2010 Medicare Physician Fee Schedule Payment Schedule published by the Centers for Medicare and Medicaid Services (Table 1).

**Table 1:** **Unit prices used to value the different types of services in the analysis (in 2010 $)**

**Service**	**Unit**	**Unit cost ($)**
Primary care physician	visit	116
Nurse practitioner	visit	87
Specialist	visit	147
Physical/Occupational therapist	visit	87
Social worker	visit	73
Homeopath/Acupuncturist	visit	59
Dentist	visit	147
Emergency room	visit	638
Hospital	visit	1916
Prescription medication	count	31
MRI	count	401
CT	count	220
Ultrasound	count	50
X-ray	count	76
Blood test	count	34
Child/personal care	hour	10
Hourly wage	hour	21

Although informal caregivers are not paid for their inputs, there is still a cost involved from the societal perspective when other opportunities are forgone. It is assumed that the work provided by informal caregivers will be similar to that of home care workers. Therefore, we used the national average hourly wage of home health and personal care aides from the 2010 Occupational Employment and Wage Estimates produced by the Bureau of Labor Statistics (BLS) to approximate the unit cost of informal caregivers as well as unpaid help by family and friends.

The days of lost work were valued using average wages obtained from the U.S. Census Bureau (U.S. Census Bureau, Statistical Abstract of the United States, 2012). We calculated daily wages from annual wages and then estimated the total lost income for each patient as a product of the total number of days missed work and daily wages. For participants who did not work, we used ½ wage rates as estimates of lost productivity [29]. Because the treatment phase for all patients began in 2009 and ended in 2011, we used 2010 prices and did not adjust for inflation [32]. For each patient, total health care expenditures were calculated as the sum of the volume of various services multiplied by the corresponding unit costs.

### Analysis

#### Outcomes

Statistical analyses were performed using STATA (Version 11, College Station, TX). We first compared patients’ baseline characteristics in the FSM and UC groups using appropriate tests of statistical significance (i.e. Chi-square test for binary variables, *t*-test for continuous variables). Last observation carried forward (LOCF) method was used to impute the 12-month outcome data for 26 individuals who did not complete the 12-month assessment and no cost data was imputed. For the effectiveness measure, we used the difference-in-difference approach in multivariate regression analysis to identify the effects of the intervention by controlling for baseline effectiveness or cost measures, as well as baseline patient characteristics (age, gender, education, marital status, employment status, number of chronic conditions, and number of symptoms).

For the cost measure, our primary interest was to examine the between-group differences in total health care expenditures among participants in the FSM as compared to those in the UC group. Therefore, we estimated total expenditures using a generalized linear model (GLM) with a gamma distribution and log link function to account for the distributional characteristics of expenditure data. We chose GLM over ordinary least squares models (with log-transformed dependent variables) based on the modified Park Test examining the distributional characteristics of residuals from both approaches as suggested by Manning and Mullahy [22,34].

### Cost-effectiveness analysis

ICERs were estimated using the standard formula: *ICER* = (*ΔC*_1_ − *ΔC*_2_)/(*ΔE*_1_ − *ΔE*_2_), where *ΔC*_1_ − *ΔC*_2_ is the difference in the average cost change from baseline to 1-year follow-up between two groups and (*ΔE*_1_ − *ΔE*_2_) is the difference in the average effectiveness change between the two groups [35]. We plotted a cost-effectiveness plane (with a cost dimension and a FSS dimension) to show the incremental change in FSS scores and in costs for FSM versus UC. The plane is divided into four quadrants: northeast (more effective, more costly), northwest (less effective, more costly), southwest (less effective, less costly), and southeast (more effective, less costly). To account for uncertainty involved in the statistical inference, 3000 incremental cost-effectiveness values were obtained through bootstrapping, a non-parametric method of statistical inference in which the empirical sampling distribution is estimated by repeated re-sampling from the observed distribution [36]. To evaluate the potential impact of imputation on ICER, plots from both the imputed sample and the complete case analysis were generated.

Because negative ICER may result in ambiguity as to which group is dominated, we used the net-benefit approach to evaluate the cost-effectiveness of the treatment group as suggested in the literature [37-40]. The net benefit approach can be defined as: *NMB* = *R*_*T*_*ΔE* − *ΔC*, where NMB = Net Monetary Benefit, *R*_*T*_ =Threshold of Willingness-to-pay per unit of benefit, *ΔE* =difference in effectiveness (net reduction in FSS score), and *ΔC* =difference in cost. Given a certain level of willingness-to-pay (often unknown from the societal perspective), NMB measures the net benefit the decision-maker is willing to pay per unit of increased effectiveness (*R*_*T*_), less the increase in cost (*ΔC*). As a result, a program is deemed cost-effective if NMB > 0 [32]. In the present study, net benefits were calculated for each patient in the sample using a range of values ($0 to $10000 in $50 increments) for *R*_*T*_ to reflect the uncertainty regarding the societal willingness-to-pay per unit of effectiveness. We then compared differences in net benefits between FSM and UC using bootstrapped multiple regression models controlling for patient characteristics and pre-treatment FSS and costs.

### Sensitivity analysis

To test the robustness of the results, we conducted sensitivity analyses under two conservative scenarios. First, because the cost of informal care is likely to be excluded from the total cost in the employer’s decision-making process of whether to adopt the intervention, we calculated the alternative total costs by assuming that the unit cost of informal care equals to zero. Second, as there is some uncertainty regarding the cost of the FSM intervention, we also calculated total costs assuming the intervention costs are 100 percent higher than our estimates. Results from this analysis will show whether the main findings are sensitive to changes in intervention costs.

## Results

### Sample characteristics

**Figure 1:**
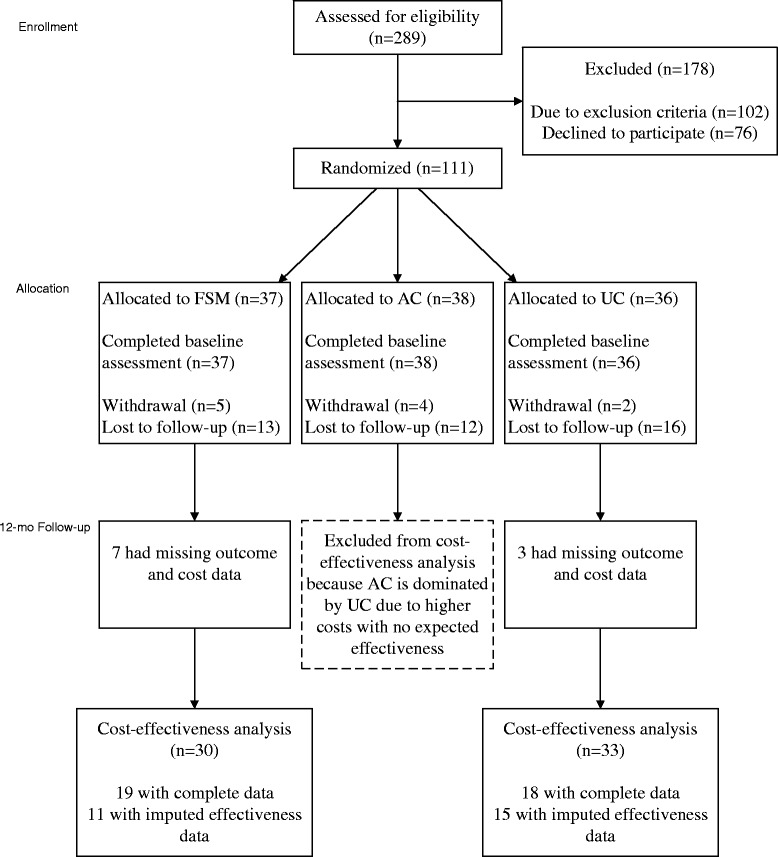
**CONSORT flow diagram.**

Although 75 individuals were randomized into the FSM (n = 37) and UC (n = 36) groups, the complete-case cost-effectiveness analysis excluded 26 individuals due to missing both effectiveness and costs data (Figure 1). Patients in FSM and UC groups did not differ significantly in baseline patient characteristics, nor are those included in the cost-effectiveness analysis differ significantly from those excluded (data not shown).

Table 2 presents average use of services and average costs by resource use categories for both groups during the study period. Overall, the FSM group had lower unadjusted average annual total cost as compared to the UC group before intervention ($3026 vs. $4862) and after the intervention ($4039 vs. $6903). As a result, the FSM group had smaller increase in average annual total costs over the study period ($1012 vs. $2041) even after the intervention costs were factored in. In terms of effectiveness, the FSM group had bigger reduction in FSS score as compared to the UC group (0.99 vs. 0.26). It appears that patients in the FSM group had smaller increases in provider visits, larger decreases in ER/hospital visits and absence from work. In summary, the unadjusted analysis showed that on average, the FSM group had better outcome and smaller increase in costs as compared to the UC group.

**Table 2:** **Costs and changes in costs for UC and FSM, by category and period**

		**Pre (3 months)**			**Post (12 months)**		
**Variables**	**n (%) users**	**# of Contacts ± SD** ^**†**^	**Cost ($)** ^**†**^	**n (%) users**	**# of Contacts ± SD** ^**†**^	**Cost ($)** ^**†**^	**Changes in Costs ($)**
**Usual Care (UC), n = 33**							
1. GP visit	21 (64)	1 ± .3	70	21 (76)	0 ± .2	47	−23
2. Specialist visit	15 (45)	1 ± .5	26	15 (67)	1 ± 1.5	60	34
3. Other provider visit	11 (33)	1 ± 1.1	58	11 (67)	1 ± 1.6	93	35
4. Provider (1 + 2 + 3)	26 (79)	1 ± 1.2	97	26 (85)	3 ± 2.4	162	65
5. ER/hospital visit	2 (6)	1 ± .2	319	2 (24)	0 ± .2	260	−59
6. Rx medications	26 (79)	1 ± .6	32	26 (85)	1 ± .8	29	−3
7. Laboratory test	21 (64)	1 ± .5	44	21 (79)	1 ± .9	47	3
8. Informal care, hours	16 (48)	43 ± 38.3	427	16 (79)	33 ± 32.3	334	−93
9. Missed work, hours	13 (39)	8 ± 8.1	125	13 (55)	6 ± 4.5	91	−34
10. Total cost (4 + 5 + 6 + 7 + 8 + 9)*			1216			1726	
11. Annualized average cost			4862			6903	2041
12. Intervention cost			0			0	
13. Grand total (11 + 12)			4862			6903	2041
**Fatigue Self-Management (FSM), n = 30**							
1. GP visit	15 (50)	1 ± .4	70	15 (73)	0 ± .7	49	−21
2. Specialist visit	8 (27)	1 ± 1.7	37	8 (80)	1 ± .8	43	6
3. Other provider visit	8 (27)	1 ± .8	77	8 (63)	1 ± 1.8	83	6
4. Provider (1 + 2 + 3)	21 (70)	1 ± 1.3	93	21 (97)	2 ± 2	127	34
5. ER/hospital visit	3 (10)	0 ± 0	213	3 (20)	0 ± .1	102	−111
6. Rx medications	21 (70)	1 ± .6	30	21 (87)	1 ± .5	21	−9
7. Laboratory test	14 (47)	1 ± .4	38	14 (77)	1 ± 1.1	29	−9
8. Informal care, hours	8 (27)	27 ± 29.5	269	8 (40)	22 ± 29.5	220	−49
9. Missed work, hours	10 (33)	10 ± 7.5	166	10 (50)	5 ± 4.7	83	−83
10. Total cost (4 + 5 + 6 + 7 + 8 + 9)*			757			939	
11. Annualized average cost			3026			3754	728
12. Intervention cost^‡^			0			285	
13. Grand total (11 + 12)			3026			4039	1012

GP = General Practitioner; ER = Emergency Room; Rx = Prescription; ^†^contacts/costs were calculated among users; *Total costs during the post- period were standardized to 3-months so that the results are comparable to the pre- period.

^‡^Intervention costs included: Personnel ($69), booklet ($10), time spent ($154), and facility and other ($52).

Table 3 summarizes the regression-adjusted incremental cost, incremental effectiveness, and incremental cost-effectiveness ratios (ICERs) for FSM versus UC. The FSM appeared to be more effective in improving FSS score and was associated with somewhat lower total costs as compared to UC. Over the 12 months study period, the FSM group had an ICER of −2358 (FSM dominant). This means that compared to UC, FSM intervention generated a societal saving of $2358 for each point reduction in the FSS score. When the bootstrapped 95% confidence interval for the ICER is considered, results suggest that the favorable ICER results for FSM should be interpreted as preliminary evidence because zero was included in the confidence interval. The same analysis using only the complete cases yielded similar results with somewhat smaller savings with wider confidence intervals, as expected from a smaller sample.

**Table 3:** **Adjusted incremental costs, effectiveness, and cost-effectiveness ratios**

**Intervention**	**Incremental cost (95% CI)**	**Incremental effectiveness (95% CI)**	**ICER**
Imputed effectiveness data, 12 mo			
UC	Reference	Reference	Reference
FSM	-$1729 (−5125,1095)	0.73 (0.15, 1.42)	FSM dominant
Complete cases, 12 mo			
UC	Reference	Reference	Reference
FSM	-$1464 (−6670,3350)	1.22 (0.16,2.55)	FSM dominant

UC = Usual Care; FSM = Fatigue Self-Management; CI = Confidence Interval.

ICER = Incremental Cost-Effectiveness Ratio, in 2010 US dollars; ICER = −2358 for imputed data, and −1199 for complete cases. Because the magnitude of negative ICER do not convey the same information as positive ICER do, “FSM dominant” is reported to indicate that FSM is more effective at lower costs as compared to UC.

Effectiveness and costs were obtained from multivariate regression models adjusting for the following baseline characteristics: age gender, education, marital status, employment status, number of chronic conditions, and number of symptoms.

**Figure 2:**
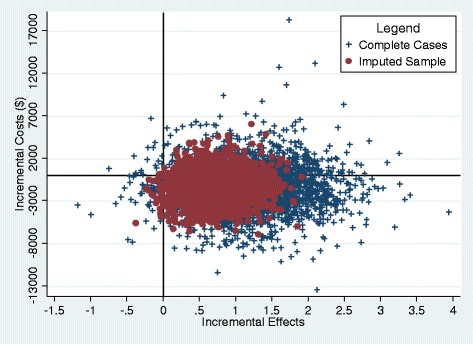
**Plots of incremental cost-effectiveness ratios for fatigue self-management and attention control from bootstrapped samples.** Note: Four quadrants: northeast (more effective, more costly), northwest (less effective, more costly), southwest (less effective, less costly), and southeast (more effective, less costly). Imputed sample included 26 individuals with imputed fatigue assessment data.

Figure 2 shows the incremental cost-effectiveness for FSM and AC as compared to UC in 3000 bootstrapped samples. Consistent with results from bivariate analysis presented in Table 2, the great majority of the ICER for FSM vs. UC fell in the southeast quadrant of the ICER plane, indicating than FSM is likely to be more effective with lower costs as compared to UC. The analysis using complete cases yielded similar results with greater uncertainty.

**Figure 3:**
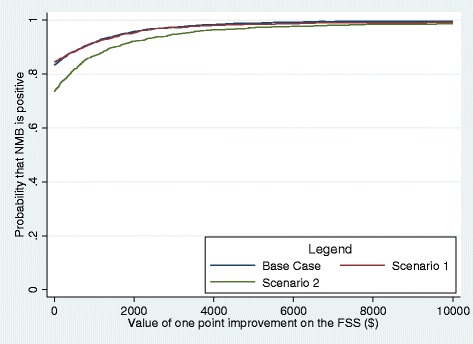
**Cost-effectiveness acceptability curve comparing fatigue self-management versus usual care, base case and sensitivity analysis.** Note: Scenario 1: informal help was valued at $0; Scenario 2: intervention cost was valued at 2 times of the base case rate.

Figure 3 presents the cost-effectiveness acceptability curve for FSM as compared to UC, as well as acceptability curves under the two scenarios of the sensitivity analysis. Scenario 1 assumes that the unit cost of informal care equals to zero and scenario 2 assumes that the intervention costs are 100 percent higher than the costs calculated in the study. For the base case, even if society values each point reduction in FSS score at $0, the probability that the FSM would generate a positive NMB is 0.833 (95% CI: 0.819, 0.847). Reducing the value of informal care had virtually no impact on NMB and doubling the intervention costs reduced the probability of positive NMB to 0.735 (95% CI: 0.710, 0.760) assuming $0 willingness-to-pay.

## Discussion

While CBT has an effect on fatigue symptoms comparable to graded exercise therapy and counseling in primary care, the longer term cost-effectiveness of CBT remains unclear [17-20]. This analysis tested the cost-effectiveness of a brief two-session CBT intervention with a self-management education component in a pilot study of primary care patients with chronic fatigue. To our knowledge, this study appears to be the first economic evaluation conducted alongside of a randomized controlled trial to examine the cost-effectiveness of a brief CBT-based FSM intervention in the U.S. The analysis of the outcome results at 12-month showed significant differences between the FSM group and the UC group after adjusting for baseline characteristics. The FSM intervention appears to be cost-effective in that it was associated with reduced fatigue impact on functioning and lower total costs.

Previous studies have shown that CBT interventions conducted in primary care by trained professionals were effective in reducing fatigue symptoms [18,19] whereas group CBT was not [15]. This study provides the first U.S. economic evidence that a brief FSM intervention may offer a promising alternative to traditional multi-session CBT delivered by experienced therapists, as it only involves two individual training sessions plus a self-help booklet, as compared to 6–16 visits in CBT trials for CFS patients [9]. This is consistent with the finding from a recent study of non-traditional face-to-face CBT, which showed that an internet-based CBT was cost-effective for severe health anxiety at 1-year follow-up [41].

A number of limitations should be considered in interpreting the findings. First, the attrition rate of this pilot study was high, suggesting that the compliance for the FSM intervention is not optimal or that the burden of monthly follow-up may be high, or both. As a result, findings such as a larger decrease in ER/hospital costs in the FSM group may due to chance. Future studies of CBT interventions should focus on better data collection techniques such as a dedicated staff person for follow-up and implementing oversight processes to reduce the amount of missing data [42]. However, given the self-management nature of this intervention, it mimics adoption rates for other behavioral interventions (such as diet and exercise) in real world settings. In addition, to the extent that the rates of attrition were similar across all three groups and that no statistical differences were detected between completers and non-completers, the attrition is unlikely to bias our findings. Nevertheless, future studies should examine what factors contribute to attrition and explore whether attrition can be reduced by modifying the intervention protocols. A second limitation is the imputation method used, which is based on the assumption that those who did not complete the study on average had no change in fatigue impact which may not be the case. However, the complete case analysis suggests that the findings were not driven by the imputation method used. Third, because patients were recruited from one geographic location, findings may not be generalizable to patients in other locations. A multi-center randomized controlled study is needed to test whether the FSM intervention is cost-effective in more diverse chronic fatigue patient populations. Finally, it is unclear to what extent the beneficial treatment effect for fatigue impact on functioning lasts beyond the 1-year follow-up period.

Despite these limitations, the present study provides important initial evidence of cost-effectiveness for a new brief two-session CBT-based FSM intervention in primary care. It has the strength of incorporating the economic data collection into the clinical outcomes measures by design and as a result, both cost and effectiveness data were collected from the same individuals [43]. Nevertheless, additional research is needed to examine how to improve treatment compliance and whether similar cost-effectiveness can be achieved in a broader patient population across multiple primary care practices and/or regions.

## Conclusion

The brief two-session CBT-based fatigue self-management intervention appeared to be cost-effective in this pilot study in that the intervention costs was more than offset by cost savings generated from reduced health services utilization during the 1 year follow-up. However, due to the small sample size and high attrition rates, interpretation of the findings should be cautious. Nevertheless, less labor-intensive modalities of CBT such as the brief nurse-led self-management approach reported here should be tested in future studies in primary care.

## Abbreviations

UC: Usual care
FSM: Fatigue self-management
CI: Confidence interval
FSS: Fatigue severity scale
ICER: Incremental cost-effectiveness ratio
NMB: Net monetary benefit

## References

[1] Whiting P, Bagnall AM, Sowden AJ, Cornell JE, Mulrow CD, Ramirez G (2001). Interventions for the treatment and management of chronic fatigue syndrome: a systematic review. JAMA.

[2] Burns D (2012). Chronic fatigue syndrome or myalgic encephalomyelitis. Nurs Stand.

[3] Chalder T, Deale A, Wessely S, Rader M, Naber D (1999). The Treatment of Chronic Fatigue Syndrome. Difficult Clinical Problems in Psychiatry. edn.

[4] Kroenke K, Wood DR, Mangelsdorff AD, Meier NJ, Powell JB (1988). Chronic fatigue in primary care. Prevalence, patient characteristics, and outcome. JAMA.

[5] Assefi NP, Coy TV, Uslan D, Smith WR, Buchwald D (2003). Financial, occupational, and personal consequences of disability in patients with chronic fatigue syndrome and fibromyalgia compared to other fatiguing conditions. J Rheumatol.

[6] Friedberg F (2010). Chronic fatigue syndrome, fibromyalgia, and related illnesses: a clinical model of assessment and intervention. J Clin Psychol.

[7] Jason LA, Taylor RR, Kennedy CL, Song S, Johnson D, Torres S (2000). Chronic fatigue syndrome: occupation, medical utilization, and subtypes in a community-based sample. J Nerv Ment Dis.

[8] McCrone P, Darbishire L, Ridsdale L, Seed P (2003). The economic cost of chronic fatigue and chronic fatigue syndrome in UK primary care. Psychol Med.

[9] Price JR, Mitchell E, Tidy E, Hunot V (2008). Cognitive behaviour therapy for chronic fatigue syndrome in adults. Cochrane Database Syst Rev.

[10] Wearden AJ, Dowrick C, Chew-Graham C, Bentall RP, Morriss RK, Peters S, Riste L, Richardson G, Lovell K, Dunn G (2010). Nurse led, home based self help treatment for patients in primary care with chronic fatigue syndrome: randomised controlled trial. BMJ.

[11] Collin SM, Crawley E, May MT, Sterne JA, Hollingworth W (2011). The impact of CFS/ME on employment and productivity in the UK: a cross-sectional study based on the CFS/ME national outcomes database. BMC Health Serv Res.

[12] White PD, Goldsmith KA, Johnson AL, Potts L, Walwyn R, DeCesare JC, Baber HL, Burgess M, Clark LV, Cox DL, Bavinton J, Angus BJ, Murphy G, Murphy M, O'Dowd H, Wilks D, McCrone P, Chalder T, Sharpe M (2011). Comparison of adaptive pacing therapy, cognitive behaviour therapy, graded exercise therapy, and specialist medical care for chronic fatigue syndrome (PACE): a randomised trial. Lancet.

[13] Moss-Morris R, Sharon C, Tobin R, Baldi JC (2005). A randomized controlled graded exercise trial for chronic fatigue syndrome: outcomes and mechanisms of change. J Health Psychol.

[14] Chambers D, Bagnall AM, Hempel S, Forbes C (2006). Interventions for the treatment, management and rehabilitation of patients with chronic fatigue syndrome/myalgic encephalomyelitis: an updated systematic review. J R Soc Med.

[15] O’Dowd H, Gladwell P, Rogers CA, Hollinghurst S, Gregory A (2006). Cognitive behavioural therapy in chronic fatigue syndrome: a randomised controlled trial of an outpatient group programme. Health Technol Assess.

[16] Twisk FN, Maes M (2009). A review on cognitive behavorial therapy (CBT) and graded exercise therapy (GET) in myalgic encephalomyelitis (ME)/chronic fatigue syndrome (CFS): CBT/GET is not only ineffective and not evidence-based, but also potentially harmful for many patients with ME/CFS. Neuro Endocrinol Lett.

[17] Chisholm D, Godfrey E, Ridsdale L, Chalder T, King M, Seed P, Wallace P, Wessely S (2001). Chronic fatigue in general practice: economic evaluation of counselling versus cognitive behaviour therapy. Br J Gen Pract.

[18] McCrone P, Ridsdale L, Darbishire L, Seed P (2004). Cost-effectiveness of cognitive behavioural therapy, graded exercise and usual care for patients with chronic fatigue in primary care. Psychol Med.

[19] Severens JL, Prins JB, van der Wilt GJ, van der Meer JW, Bleijenberg G (2004). Cost-effectiveness of cognitive behaviour therapy for patients with chronic fatigue syndrome. QJM.

[20] Sabes-Figuera R, McCrone P, Hurley M, King M, Donaldson AN, Ridsdale L (2012). Cost-effectiveness of counselling, graded-exercise and usual care for chronic fatigue: evidence from a randomised trial in primary care. BMC Health Serv Res.

[21] Yates BT (1994). Toward the incorporation of costs, cost-effectiveness analysis, and cost-benefit analysis into clinical research. J Consult Clin Psychol.

[22] Doshi JA, Glick HA, Polsky D (2006). Analyses of cost data in economic evaluations conducted alongside randomized controlled trials. Value Health.

[23] Friedberg F, Napoli A, Coronel J, Adaomwicz J, Seva V, Caikauskaite I, Ngan M, Chang J, Meng H (2013). Chronic fatigue self management in primary care: a randomized trial. Psychosom Med.

[24] First MB, Spitzer R, Gibbon M, Willams J (2001). Structured Clinical Interview for DSM-IV Axis I Disorders (SCID-I).

[25] Schulz KF, Altman DG, Moher D (2010). CONSORT 2010 statement: updated guidelines for reporting parallel group randomized trials. Ann Intern Med.

[26] Jason L, Torres-Harding S, Friedberg F, Corradi K, Njoku M, Donalek J, Reynolds N, Brown M, Weitner B, Rademaker A, Papernik M (2007). Non-pharmacologic interventions for CFS: a randomized trial. J Clin Psychol Med Settings.

[27] Krupp LB, LaRocca NG, Muir-Nash J, Steinberg AD (1989). The fatigue severity scale. Application to patients with multiple sclerosis and systemic lupus erythematosus. Arch Neurol.

[28] Taylor RR, Jason LA, Torres A (2000). Fatigue rating scales: an empirical comparison. Psychol Med.

[29] Gold M, Siegel J, Russell L, Weinstein M (1996). Cost-Effectiveness in Health and Medicine.

[30] Beecham J, Knapp M, Thornicroft G (2001). Costing Psychiatric Interventions. Measuring Mental Health Needs.

[31] Goossens MEJB, Rutten-Van Mölken MPMH, Kole-Snijders AMJ, Vlaeyen JWS, Van Breukelen G, Leidl R (1998). Health economic assessment of behavioural rehabilitation in chronic low back pain: a randomised clinical trial. Health Econ.

[32] Glick HA, Doshi JA, Sonnad SS, Polsky D (2007). Economic Evaluation in Clinical Trials.

[33] Oostenbrink JB, Koopmanschap MA, Rutten FF (2002). Standardisation of costs: the Dutch Manual for Costing in economic evaluations. PharmacoEconomics.

[34] Manning WG, Mullahy J (2001). Estimating log models: to transform or not to transform?. J Health Econ.

[35] Drummond M, Sculpher M, Torrance G, O’Brien B, Stoddart G (2005). Methods for the Economic Evaluation of Health Care Programmes.

[36] Mooney CZ (1996). Bootstrap statistical inference: examples and evaluations for political science. Am J Polit Sci.

[37] Briggs AH, O’Brien BJ, Blackhouse G (2002). Thinking outside the box: recent advances in the analysis and presentation of uncertainty in cost-effectiveness studies. Annu Rev Public Health.

[38] Stinnett AA, Mullahy J (1997). The negative side of cost-effectiveness analysis. JAMA.

[39] Briggs AH (2001). A Bayesian approach to stochastic cost-effectiveness analysis. An illustration and application to blood pressure control in type 2 diabetes. Int J Technol Assess Health Care.

[40] Richardson G, Epstein D, Chew-Graham C, Dowrick C, Bentall RP, Morriss RK, Peters S, Riste L, Lovell K, Dunn G, Wearden AJ, FINE Trial Writing group on behalf of the FINE Trial group (2013). Cost-effectiveness of supported self-management for CFS/ME patients in primary care. BMC Fam Pract.

[41] Hedman E, Andersson E, Lindefors N, Andersson G, Ruck C, Ljotsson B (2013). Cost-effectiveness and long-term effectiveness of internet-based cognitive behaviour therapy for severe health anxiety. Psychol Med.

[42] Fleming TR (2011). Addressing missing data in clinical trials. Ann Intern Med.

[43] Ramsey S, Willke R, Briggs A, Brown R, Buxton M, Chawla A, Cook J, Glick H, Liljas B, Petitti D, Reed S (2005). Good research practices for cost-effectiveness analysis alongside clinical trials: the ISPOR RCT-CEA Task Force report. Value Health.

